# Collaborative Networks in Critical Care Nursing Teams: Based on Social Network Analysis and Multiple Regression Quadratic Assignment Procedure

**DOI:** 10.1155/jonm/6045308

**Published:** 2026-06-18

**Authors:** Jiaqi Shi, Qingxia Quan, Kaili Ye, Lulu Feng, Fangfang Chen, Jianghui Yang, Zhenhong Fang

**Affiliations:** ^1^ Department of Intensive Care Unit, The First Affiliated Hospital of Wenzhou Medical University, Wenzhou, Zhejiang, 325015, China, wzhospital.cn; ^2^ School of Information and Engineering, Wenzhou Medical University, Wenzhou, Zhejiang, 325035, China, wmu.edu.cn

**Keywords:** critical care nursing, nurse collaboration, social network analysis

## Abstract

**Background:**

Effective collaboration among intensive care unit (ICU) nurses is critical for patient safety, yet traditional analyses often overlook the relational dependencies inherent in teamwork. This study aimed to delineate task‐specific collaboration structures and identify pair‐level determinants using network‐aware methodologies.

**Methods:**

A cross‐sectional study was conducted in a tertiary ICU involving 96 registered nurses during day shifts from January 1, 2025, to February 1, 2025. Collaboration was defined as coparticipation in clinical events and categorized into four task types: emergent response, procedural care, patient flow, and safety double‐checks.

**Results:**

Event‐based networks revealed distinct topologies: Emergent care showed compact clustering, while procedural care displayed hub‐and‐spoke configurations centered on specialists. MRQAP analysis (*R* ^2^ = 0.27) identified work assignment proximity as the strongest predictor of collaboration (*β* = 0.314, *p* < 0.001). Psychological safety similarity (*β* = 0.176, *p* = 0.004) and competency complementarity (*β* = 0.142, *p* = 0.021) were also significant, while tenure similarity showed a borderline association.

**Conclusions:**

ICU nurse collaboration is multidimensional and task‐dependent. Managerial strategies should prioritize optimizing roster designs for spatial proximity, fostering a psychologically safe climate, and strategically developing complementary competencies to enhance team resilience and care quality.

## 1. Introduction

Intensive care units (ICUs) are the hospital’s core clinical hubs, caring for the most critically ill patients in the most technology‐dense and time‐pressured settings. Nursing work in this environment extends well beyond routine care and into advanced life support activities that hinge on tight coordination. Day‐to‐day practice includes highly specialized procedures, such as continuous renal replacement therapy (CRRT) management and artificial airway maintenance, while moment‐to‐moment responses encompass resuscitation and the verification of high‐alert medications. These tasks share three traits—high interdependence, narrow time windows, and substantial risk—and their safe, effective execution depends fundamentally on precise and efficient nurse‐to‐nurse collaboration [1]. A large body of evidence links the quality of ICU teamwork to rates of adverse events, including catheter‐related infections and unplanned extubations, and to both resuscitation success in critically ill patients and levels of nursing burnout [2, 3]. Accordingly, understanding and systematically optimizing patterns of collaboration among nurses has become a central route to improving ICU care quality and patient safety.

Yet, the construction and maintenance of efficient collaboration in everyday ICU practice are constrained by deep, systemic realities. Foremost is the global shortfall in nursing personnel, a structural pressure that collides with high‐intensity workloads [4, 5]. Frequent shift rotations further fragment the temporal and spatial continuity of teamwork, making it hard for stable partnerships to form and mature. These constraints do not merely lower the frequency of interaction or slow the growth of tacit coordination; they also erode the completeness of information transfer and the timeliness of decision support, with downstream effects on patient outcomes [5]. Within fixed staffing levels and rotation schedules, the practical management question becomes how to extract the most from existing conditions and redesign collaboration structures for maximal effect.

In recent years, scholarship has moved beyond simple task allocation to foreground the role of intrateam collaboration, with growing attention to psychological safety, unit climate, and related organizational factors that shape how nurses work together [6, 7]. Even so, the evidence base remains dominated by cross‐sectional designs that rely on conventional regression to probe correlates of collaboration [3]. Such models rest on an independence assumption among individuals. Collaboration, by contrast, is intrinsically relational: Behavior in one nurse pair is linked to behavior in others, and the data carry built‐in interdependence. Standard regression therefore struggles to uncover structural determinants embedded in collaboration networks and can generate biased or misleading inferences. These constraints leave our understanding of ICU nurse‐to‐nurse collaboration incomplete and point to the need for analytic approaches that align with the relational nature of teamwork.

Social network analysis (SNA) offers a structured lens on collaborative ties, mapping the overall interaction pattern through familiar metrics, such as network density, centrality, and community structure [8]. Unlike individual‐level approaches, it foregrounds relational dependence and can reveal which nurses act as hubs or bridges—closer to the lived mechanics of collaboration in practice [9]. Building on that foundation, the multiple regression quadratic assignment procedure (MRQAP) brings regression into the network setting by using permutation tests to honor the dependence inherent in relational data. The approach estimates the effects of multiple predictors on collaboration while avoiding the bias that arises when nonindependence is ignored, making it especially well‐suited to identify structural determinants in ICU nurse collaboration networks.

Despite growing international attention to team collaboration, systematic research on ICU nurse collaboration networks remains scarce. Much of the existing work is cross‐sectional and largely descriptive, offering limited insight into the structural drivers of collaboration; methodologically, few studies in nursing have combined SNA with MRQAP. Although multidisciplinary teamwork is fundamental to ICU care, nurses constitute the largest continuously present professional group in the ICU and are centrally involved in day‐to‐day coordination across monitoring, treatment implementation, patient flow, procedural support, and safety verification. Accordingly, the present study intentionally defined the network boundary at the intranursing level in order to examine collaboration structures within a relatively homogeneous professional context. As a result, our understanding of ICU nurse‐to‐nurse collaboration is still incomplete. Against this backdrop, we employ SNA and MRQAP to conduct an in‐depth case study of one tertiary ICU, delineating task‐specific collaboration structures and examining how work assignment proximity, psychological safety, competency complementarity, and tenure relate to collaboration strength. Findings are interpreted in light of the specific institutional setting, with the goal of providing contextually grounded evidence to inform team development and managerial practice in critical care nursing.

## 2. Materials and Methods

### 2.1. Study Design and Sample

This cross‐sectional study was conducted in a large tertiary‐level teaching hospital operating under an open ICU model, with 50 fixed ICU beds providing continuous care for critically ill patients. The ICU nursing workforce consists of 113 registered nurses directly engaged in bedside and patient‐related care, including auxiliary staff and administrative managers. Data were collected from day shifts (08:00–16:30), January 1, 2025, to February 1, 2025. Inclusion criteria include the following: ① registered nurses formally assigned to the ICU roster during the observation window; ② at least one day‐shift presence (08:00–16:30) on the ward within the observation period; and ③ engagement in patient‐facing clinical duties, including bedside care, treatment or transport tasks, or charge nurse roles physically present on the ward. Exclusion criteria include the following: ① nurse managers, educators, or administrative staff whose duties were exclusively nonclinical; ② temporary, float, or agency nurses not included in the regular ICU roster; ③ nursing students, interns, or trainees without RN licensure; and ④ nurses absent for the entire observation window, or on leave for more than half of the window with no day‐shift presence. The final analytic sample included 96 registered nurses, and this full set served as the node list for network analyses.

### 2.2. Data Sources and Quality Control

Data were extracted from the electronic rostering system and the nursing documentation system. The extract covered on‐duty records, patient assignment, and responsibility allocation for the entire window. Clinical collaboration events were captured through two complementary sources. Structured data—including on‐duty records, patient assignments, and co‐signed nursing documentation—were extracted from the electronic rostering and nursing information systems. In parallel, trained research observers conducted nonparticipatory, real‐time observation during day shifts to record coparticipation in qualifying clinical events, supplementing electronic records where system documentation was incomplete. These observers were members of the research team but were not involved in ICU staffing management or personnel evaluation. Observer logs and electronic records were cross‐referenced and reconciled through a dual‐review workflow. This triangulated approach aimed to maximize event capture while minimizing reliance on any single data source. Person identifiers and timestamps were harmonized across systems. Entries referring to the same patient, the same event type, and the same calendar date were collapsed using a deterministic de‐duplication key. Only events verified to have occurred during day shifts with all participants on duty were retained. A dual‐review workflow and random audits ensured consistency, and all missing or conflicting entries along with their resolutions were logged.

### 2.3. Definition of Collaboration

Guided by ICU operations standards [10–13] and a structured expert consultation, we classified common ICU collaboration tasks into four analytic categories: emergent response, procedural or technical team nursing, patient flow and handover, and safety‐critical double‐checks:

Type 1: Emergent/acute care, including cardiopulmonary resuscitation (CPR), airway management/urgent intubation, massive transfusion, and bedside defibrillation;

Type 2: Procedural/teamwork care, including CRRT setup/connection/maintenance; tracheostomy care/fixation; bedside bronchoscopy assistance; and basic nursing care combining repositioning with two‐person hygiene;

Type 3: Patient flow and handover, including admission to ICU, intrahospital transport, and postoperative handover;

Type 4: Safety double‐checks, including psychotropic medication verification, narcotic medication verification, high‐alert medication double‐checks, and blood transfusion double‐checks.

Events served as the unit of analysis. For each event, we identified the nurses who participated during the same day shift and linked each nurse pair once, treating pairs as unordered. This created an undirected edge with weight 1 and attributes: date, shift, category, subtype, and a de‐identified patient code. Multinurse events generated all unique nurse pairs exactly once; for example, one CPR performed by nurses A, B, and C yielded A–B, A–C, and B–C. Records that referred to the same patient, the same event category, and the same calendar date were collapsed to a single count per nurse pairs. An edge was created only when both nurses were documented on duty for the same day shift; contacts across shifts or across days were not counted as collaboration. The event‐level edge list was retained for analysis to preserve timing and heterogeneity. For descriptive summaries and figures, we aggregated by nurse pairs across the observation window to obtain *Y*_*i* *j*_. Thus, the analytical unit of this study was the pairwise coparticipation tie rather than the individual nurse‐day. The reported collaboration counts therefore represent predefined event‐based nurse pair ties occurring during eligible day shifts, rather than the total volume of all nurse‐to‐nurse interactions in the ICU. Collaboration opportunity was quantified as *C*_*i* *j*_, the number of day shifts on which both nurses were simultaneously on duty.

### 2.4. Measurements

#### 2.4.1. General Characteristics of Participants

Demographic and professional information was collected for all nurses included in the study. Variables comprised age, gender, educational background, professional title, ICU tenure, and training certifications: advanced cardiovascular life support, CRRT, and mechanical ventilation training.

#### 2.4.2. Selection of Explanatory Variables

A comprehensive pool of candidate variables was initially developed from prior research on ICU teamwork and SNA, covering structural, individual, relational, and competency‐related domains [14–16]. This candidate pool was then subjected to expert screening, where senior ICU nurses and nurse managers assessed each variable for scientific relevance and feasibility of data collection. Based on this evaluation, six variables were retained for further consideration: work assignment proximity, psychological safety similarity, informal subgroup comembership, competency complementarity, tenure similarity, and patient acuity overlap. The full list of candidates, together with reasons for inclusion or exclusion, is provided in Supporting Table S1. Each of the retained variables is described below, along with its conceptual definition and data source.

1. Work Assignment Proximity: This variable captured whether two nurses were assigned to the same responsibility unit during a day shift, as recorded in official rosters. Being co‐assigned reflects structural opportunities and expectations to collaborate. Data were aggregated across the observation window to represent the frequency of shared assignments.
2. Psychological Safety Similarity: Psychological safety similarity was assessed using Edmondson’s 7‐item Team Psychological Safety Scale [17]. Each nurse completed the instrument on a 5‐point Likert scale (1 = *strongly disagree*; 5 = *strongly agree*), with higher scores indicating greater perceived psychological safety. Pairwise similarity between nurses’ scores was subsequently derived to capture pair‐level resemblance.
3. Informal Subgroup Comembership: Informal subgroup comembership was assessed through a brief self‐administered questionnaire included in the same survey as the psychological safety scale. Nurses were asked whether they perceived themselves as belonging to a fixed informal peer group within the ICU (yes/no), and if so, to indicate the main members of that group. Nurse pairs were coded as comembers if both nurses reported belonging to the same informal group.
4. Competency Complementarity: This variable assessed whether two nurses possessed complementary certifications enabling joint execution of specialized or high‐risk tasks. Certification records were obtained from hospital training logs, including competencies, such as ACLS, CRRT, ventilator management, transfusion verification, and bronchoscopy assistance. Nurse pairs were identified as complementary if their combined certifications fulfilled predefined task requirements.
5. Tenure Similarity: Tenure similarity represented the closeness of ICU experience in years, based on human resources records. Nurse pairs with smaller differences in ICU tenure were considered more similar, reflecting shared tacit knowledge and routines. This measure was used to test whether experiential homogeneity facilitates collaboration.
6. Patient Acuity Overlap: Patient acuity overlap captured whether two nurses simultaneously cared for high‐acuity patients within the same day shift. Unit‐level acuity was derived from patient monitoring and admission records. Nurse pairs with a higher proportion of copresence in high‐acuity contexts were considered to have greater overlap, reflecting increased likelihood of collaboration under critical clinical demands.

These six variables were subsequently entered into a Least Absolute Shrinkage and Selection Operator (LASSO) regression with the collaboration matrix (Y) as the dependent variable. Details of the LASSO procedure and the operationalization of the final selected variables are presented in Section 2.5.3.

### 2.5. Data Analysis

#### 2.5.1. SNA

**FIGURE 1:**
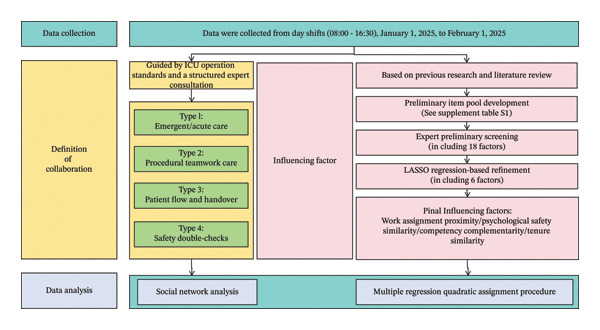
Analytical framework for this study.

The overall analytical workflow is illustrated in Figure 1, summarizing the process from data collection and task definition to factor identification and network‐based analysis. ICU collaboration was represented as a weighted and undirected network. Nodes were registered nurses. An edge linked a nurse pair when they worked together in a qualifying event during the same day shift. Edge weight was the number of such events across the observation window, written as *Y*_*i* *j*_. Pairs were treated as unordered, so *i* with *j* equals *j* with *i*. We described the network with density, mean degree, weighted degree and betweenness centrality, the clustering coefficient, and a modularity‐based community view. Visual maps used a force‐directed layout; node size reflected weighted degree, edge thickness reflected weight, and node color indicated communities. All network figures and community detection were produced in Gephi. This descriptive map sets the scene for which pair‐level factors to test next and gives clinical context for the regression that follows.

#### 2.5.2. Predictor Screening With LASSO

Six candidate variables were screened using 10‐fold cross‐validation LASSO regression with the collaboration matrix as the outcome. Predictors with nonzero coefficients at the optimal penalty were carried forward to MRQAP. Full regularization paths and cross‐validation results are provided in Supporting Figure S1.

#### 2.5.3. MRQAP

Using the predictors retained above and the same exposure adjustment, we estimated associations with a network‐aware regression using MRQAP, with *Y*_*i* *j*_ as the outcome and shared day shifts kept as an exposure term. MRQAP was chosen because nurse pairs are not independent. Each nurse appears in many pairs, and ordinary regression would underestimate uncertainty. MRQAP uses permutation tests that respect network structure, which keeps *p* values valid under this dependence. Results are reported as standardized coefficients with network‐corrected *p* values and clear statements of direction and practical meaning. This final step turns descriptive patterns into estimates that account for network structure while staying comparable across predictors.

### 2.6. Ethical Considerations

The study protocol underwent prereview approval by the First Affiliated Hospital of Wenzhou Medical University in December 2024 (Prereview Approval No. YS2024‐1005), before the initiation of data collection. Final ethics approval was subsequently obtained from the Institutional Review Board of The First Affiliated Hospital of Wenzhou Medical University in April 2026 (Approval No. KY2026‐168). All participating nurses received an information sheet explaining the study purpose, voluntary participation, and data confidentiality. Written informed consent was obtained before survey administration. Identifiable information was de‐identified before analysis, and network visualizations were anonymized to prevent disclosure of individual identities. The study adhered to the principles of the Declaration of Helsinki.

## 3. Results

### 3.1. Descriptive Statistics

The sample comprised 96 ICU nurses with a mean age of 30.15 ± 4.99 years. Education was predominantly the bachelor level, 90.6%. Professional titles were mainly junior, 83.3%. ICU tenure clustered at ≤ 3 years, 41.7%. Full details are shown in Table 1.

**TABLE 1:** Demographic and professional characteristics of ICU nurses.

Variables	Subgroup	*n* = 96 (%)
Age	($\overset{¯}{x}$ ± *s*)	(30.15 ± 4.99)

Gender	Male	14 (14.6%)
	Female	82 (85.4%)

Educational background	Associate degree	6 (6.3%)
	Bachelor	87 (90.6%)
	Master and above	3 (3.1%)

Professional title	Junior	80 (83.3%)
	Intermediate	14 (14.6%)
	Senior	2 (2.1%)

ICU tenure	≤ 3 years	40 (41.7%)
	4–7 years	31 (32.3%)
	≥ 8 years	25 (26.0%)

Training certifications	Advanced cardiovascular life support	8
	Continuous renal replacement therapy	82
	Mechanical ventilation training	7

*Note:* Training certifications were multiple‐response items; totals may exceed or fall below 96 due to multiple qualifications and missing data.

### 3.2. Predictor Selection Using LASSO Regression

LASSO retained four predictors: work assignment proximity, psychological safety similarity, tenure similarity, and competency complementarity. Informal subgroup comembership and patient acuity overlap were reduced to zero and excluded from subsequent analyses. Details of the regularization paths and cross‐validation curve are provided in Supporting Figure S1.

### 3.3. SNA Results

**FIGURE 2:**
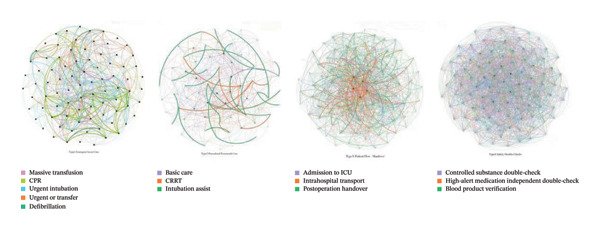
Event‐based collaboration networks (Type 1–Type 4). From left to right: Type 1 (emergent care), Type 2 (procedural/teamwork), Type 3 (patient flow/handover), and Type 4 (safety double‐checks).

Across all four collaboration types, a total of 4045 coparticipation events were recorded, each representing a documented instance of two nurses working together on the same clinical task during the same shift. These events were mapped as collaboration networks, where each dot represents a nurse, and each line connecting two dots represents at least one documented collaboration between them. The thickness of the line reflects how frequently that pair worked together. As shown in Figure 2 and summarized in Table 2, the four task‐specific networks display strikingly different patterns. In emergent care (Type 1), the network is relatively sparse (17% of all possible nurse pairs collaborated), with collaboration concentrated within small, tightly connected groups—reflecting the reality that resuscitation and airway emergencies are handled by a compact team that rapidly converges at the bedside. In procedural and technical care (Type 2), the network is the most sparse of all (12%), with a clear “hub‐and‐spoke” shape: A small number of specialist nurses—such as those certified in CRRT or airway management—appear as central hubs connected to many colleagues, while most nurses connect primarily through these specialists. In patient flow and handover (Type 3), collaboration is more broadly distributed (22%), with certain nurses—such as transport nurses and charge nurses—acting as bridges linking different parts of the team. Finally, the safety double‐check network (Type 4) is the most densely connected (35%), reflecting the mandatory dual‐verification requirement for high‐risk medications and blood products, which necessitates that almost every nurse pairs with a wide range of colleagues throughout the shift.

**TABLE 2:** Global network metrics for event‐based collaboration subnetworks.

Category	Nodes	Edges	Density	Avg degree
Type 1	84	591	0.17	14.07
Type 2	81	376	0.12	9.28
Type 3	96	1002	0.22	12.88
Type 4	96	2076	0.35	16.25

### 3.4. MRQAP Results

Guided by the LASSO screen, we estimated MRQAP with the four retained predictors. Work assignment proximity showed the largest effect (*β* = 0.314, *p* < 0.001); psychological safety similarity (*β* = 0.176, *p* = 0.004) and competency complementarity (*β* = 0.142, *p* = 0.021) were also significant; tenure similarity was borderline (*β* = 0.097, *p* = 0.062). This concordance in signs and significance provides inferential support for the LASSO selection, while the model fit (*R* ^2^ = 0.27) indicates moderate explanatory power. Full estimates are reported in Table 3.

**TABLE 3:** MRQAP results for LASSO‐selected predictors of nurse‐to‐nurse collaboration.

Variable	Standardized *β*	95% CI	*p*‐value
Work assignment proximity	0.314	(0.198, 0.431)	< 0.001
Psychological safety similarity	0.176	(0.058, 0.294)	0.004
Competency complementarity	0.142	(0.022, 0.263)	0.021
Tenure similarity	0.097	(−0.005, 0.199)	0.062
Model *R* ^2^	0.27	—	—

*Note:* MRQAP analysis based on 5000 permutations. The collaboration matrix was symmetric and undirected. All explanatory matrices were standardized.

## 4. Discussion

Intensive care nursing is technically demanding, time‐critical, and risk‐laden; breakdowns in collaboration bear directly on resuscitation outcomes and on medication and transfusion safety. Staffing constraints and frequent rotations fragment collaboration opportunities, limiting information continuity and slowing decisions, which underscores the need to depict ICU nurse collaboration in real clinical contexts. Accordingly, we used event‐based SNA to model internurse ties as coparticipation in care events and applied MRQAP to test whether assignment proximity, similarity in psychological safety, complementary competencies, and similarity in tenure were associated with collaboration strength. The collaboration landscape comprised four event‐specific networks, and the analysis identified factors shaping their strength and topology. Methodologically, rather than treating collaboration as an isolated individual attribute, the approach conceptualizes it as a relation generated by shared events and conducts inference while respecting network dependence, yielding a portrayal closer to how teams actually work. In practice, these findings offer tentative, actionable leads for modest adjustments to rostering and zoning, more deliberate handover refinement, and a more balanced distribution of load at key positions, thereby providing an empirical basis for targeted interventions and continuous quality improvement.

Event‐based network analysis revealed marked heterogeneity in ICU nurse collaboration structures, closely mirroring the intrinsic logic of distinct clinical task types. In emergent response scenarios (Type 1), the network exhibited a compact, clustered morphology with low overall connection density (density = 0.17), accurately reflecting the operational pattern of critical events, such as CPR—where collaboration rapidly converges within small, highly mobile “action pods” [18]. Time pressure, interchangeability of roles among core team members, and spatial colocation at the bedside reinforced strong intraunit cohesion but constrained cross‐unit linkages, resulting in high local clustering, transient yet intense interactions, and a moderate overall network reach (average degree = 14.07). From a managerial perspective, this pattern suggests the value of designating relatively stable core resuscitation teams for each shift. Brief cross‐unit coordination drills during safety handover meetings could further enhance interunit alignment. In addition, appointing rotating liaisons within each responsibility unit to monitor and communicate cross‐unit needs may help bridge collaboration gaps without substantially increasing workload [19].

In procedural or technical care (Type 2), the network displayed a pronounced hub‐and‐spoke configuration and the lowest overall connection density (density = 0.12). Although CRRT certification was widely distributed among ICU nurses, CRRT initiation and other high‐frequency procedural tasks during day shifts were mainly undertaken by a smaller group of highly experienced CRRT expert nurses because CRRT‐related treatments were relatively concentrated during daytime working hours. These nurses therefore emerged as central hubs connected to multiple colleagues through repeated participation in specialized procedural workflows. This pattern underscores the functional value of professional specialization: Complex techniques, such as CRRT, are often concentrated among highly experienced operators, which may enhance procedural standardization and operational efficiency [20, 21]. However, such centralization, while advantageous for technical excellence, simultaneously introduces latent risks by creating deep dependency on these hub nodes—overload or absence of a core specialist may generate collaboration bottlenecks and compromise service continuity. Accordingly, managerial strategies should adopt a dual‐track approach: first, to fully leverage the expertise of specialist nurses while systematically identifying these pivotal roles and establishing small standby pools with basic cross‐training [22, 23]; and second, at the equipment‐deployment level, to position “satellite” device packs in key zones and institute secondary backup mechanisms. These measures can disperse single‐point‐failure risk while maintaining workflow efficiency, ensuring the robustness and sustainability of specialized nursing support [20, 24].

In patient flow and handover (Type 3), the network exhibited moderate connection density (density = 0.22) and was characterized by distinct intermediary nodes—chief nurses, transport nurses, or handover coordinators—who functioned as bridges linking the network’s 96 nodes (edges = 1002). Evidence suggests that dedicated transport nurses substantially improve the efficiency and safety of intrahospital transfers for critically ill patients [25, 26], while standardized handover protocols have been shown to reduce information transfer errors [27]. This network configuration thus reinforces the value of functional specialization in enhancing clinical efficiency: Transport nurses concentrate on patient transfer to ensure continuity of care, whereas handover coordinators safeguard informational integrity through structured communication. Building on this logic, management practice should further delineate the role boundaries of these intermediary positions, institute backup mechanisms, and implement standardized handover procedures within defined time windows accompanied by concise checklists. Such measures would fully exploit the strengths of professional specialization while balancing continuity and time efficiency [28, 29].

The safety‐check network displayed the highest connection density (density = 0.35) and the broadest coverage (edges = 2076; average degree = 16.25). This diffuse structure, driven by the mandatory dual‐verification system, effectively prevents clinical errors; however, its widespread shallow linkages and frequent procedural interactions can induce “checking fatigue” [30]. To preserve safety while enhancing efficiency, a three‐tiered optimization strategy is recommended. At the technical level, verification steps should be deeply integrated into the electronic medical record system, using electronic cosignatures and similar tools to eliminate unnecessary manual steps [31]. At the managerial level, drawing on high‐reliability‐organization practices, verification items can be stratified by risk level to focus limited cognitive resources on the most critical points [32]. At the system level, complex procedures, such as blood transfusion, should be embedded within comprehensive quality control and nursing‐sensitive‐indicator systems, supported by intelligent nursing platforms enabling real‐time monitoring and closed‐loop management. Collectively, these evidence‐based optimizations constitute a coherent technical‐managerial‐system solution that sustains safety redundancy while improving clinical work efficiency [33, 34]. These findings are broadly consistent with, and extend, prior SNA‐based studies of healthcare team collaboration. Kawamoto [35] used wearable sociometric sensors in an ICU setting and similarly found that nurses constitute the central nodes of the communication network. A subsequent longitudinal study further demonstrated that changes in leadership structure altered network centrality patterns, suggesting that collaboration structures are sensitive to organizational interventions—a finding that reinforces the managerial implications of our own results [36, 37]. By categorizing collaboration into four clinically meaningful task types, our event‐based approach further reveals how network topology varies systematically with the nature of the work, offering a task‐specific perspective that complements existing interaction‐based evidence. The marked differences in density, edge distribution, and topology across the four event‐based subnetworks further suggest that collaboration mechanisms may vary substantially according to task context. Consequently, within a limited observation window, the overall network may provide a more stable representation of ICU nurse collaboration patterns than task‐specific subnetworks.

The MRQAP results indicate that ICU nurse collaboration is shaped by a multidomain configuration spanning structural, individual, relational, and competency factors. From the structural domain, work assignment proximity showed the largest association with collaboration (*β* = 0.314, *p* < 0.001), a pattern that aligns with the descriptive network maps in which most ties arise between colocated nurses on shared assignments. This finding is consistent with prior studies highlighting the importance of spatial proximity and shared task environments in facilitating coordination and communication among healthcare professionals [38]. This convergence of SNA and MRQAP emphasizes colocation and shared tasking as critical levers for everyday collaboration. In the individual domain, psychological safety similarity (*β* = 0.176, *p* = 0.004) suggests that shared perceptions of interpersonal safety enable more open communication, highlighting the role of unit climate in converting structural opportunity into effective joint action [39, 40]. The association between psychological safety similarity and collaboration warrants further interpretive attention. While this pattern may partly reflect shared work experiences shaping converging safety perceptions, it is unlikely to be explained solely by this mechanism. Psychological safety has been conceptualized as a multilevel construct, with dyadic perceptions forming an important basis for team‐level climate [17]. Emerging evidence suggests that not only the level, but also the alignment of safety perceptions between individuals may influence how effectively they communicate and coordinate [41]. In this context, our findings suggest that nurses with similar perceptions of interpersonal risk may be more likely to engage in open communication and collaborative action. Whether this similarity reflects genuine psychological alignment or is partly shaped by shared unit experiences remains an open question, warranting future dyadic and longitudinal investigation. Within the relational domain, tenure similarity exhibited a borderline association (*β* = 0.097, *p* = 0.062), indicating a tendency toward same‐experience pairing; while this may streamline coordination [42], it could dampen the flow of tacit knowledge from senior to junior staff, pointing to the value of structured mentoring and mixed‐tenure teaming [43]. Finally, in the competency domain, competency complementarity (*β* = 0.142, *p* = 0.021) was positively associated with collaboration, consistent with subnetwork topologies: The hub‐and‐spoke pattern in procedural/technical care (Type 2) concentrates activity around certified specialists (e.g., CRRT leads), whereas bridging roles in patient flow/handover (Type 3) reflect coordination‐specific competencies. This is also consistent with previous research showing that complementary expertise enhances team performance in complex clinical tasks, with studies in ICU settings demonstrating that teams where members possess distinct but mutually reinforcing skills are better equipped to manage high‐acuity care demands [44]. Taken together, these findings argue for a synergistic strategy that couples structural design, cultivation of team climate, intentional management of experience mix, and targeted development of specialized skill sets. Beyond statistical associations, it is worth situating these findings in their clinical context. To provide clinical context for interpreting these network patterns, nursing‐sensitive indicators during the observation period included an unplanned extubation rate of 2% and a medication error rate of 0.18%—both consistent with or below ranges reported in comparable tertiary ICU settings [45, 46]. While causal attribution is beyond the scope of this cross‐sectional design, these indicators suggest that the collaboration structures identified in this study operated within a functionally effective clinical environment, lending real‐world grounding to the network patterns described.

Several limitations warrant consideration. First, the study was conducted in a 50‐bed ICU within a university‐affiliated tertiary hospital in an economically developed region of eastern China, representing a relatively large and well‐resourced critical care environment. In terms of workforce structure, shift patterns, workload intensity, and task organization, it is broadly comparable to many tertiary ICUs across China, suggesting reasonable transferability of the observed collaboration patterns to similar high‐acuity settings. However, the larger bed capacity and higher resource availability may limit generalizability to smaller hospitals, rural or less‐developed regions, or healthcare systems with different organizational cultures and nurse‐to‐patient ratios. Second, the observation window was limited to January 1, 2025, to February 1, 2025; while a focused window is methodologically appropriate for capturing a stable network snapshot, seasonal variation in staffing or case mix across the year was not examined and may influence collaboration structures. Third, data collection was restricted to day shifts; although collaboration patterns during night shifts are expected to be broadly similar, the reduced staffing levels may concentrate collaborative ties among fewer nurses, a structural difference that warrants acknowledgment. Fourth, although the triangulated approach combining electronic records and nonparticipatory observation substantially improved event capture compared with single‐source methods, some informal collaboration occurring outside documented clinical events may remain undetected, and true collaboration frequency may be modestly underestimated. Fifth, the cross‐sectional design precludes causal inference, and the moderate model fit (*R* ^2^ = 0.27) indicates residual variance likely driven by unmeasured contextual factors.

## 5. Conclusion

By integrating the SNA with the MRQAP, this study systematically revealed the structural characteristics and key determinants of ICU nurse collaboration networks. Four distinct topologies—emergency response, procedural or technical care, patient flow and handover, and safety double‐checking—reflected the task‐specific nature of collaboration. MRQAP identified work assignment proximity, psychological safety similarity, competency complementarity, and tenure similarity as core factors shaping tie strength. These findings underscore the multidimensional and context‐dependent nature of ICU collaboration while demonstrating the methodological value of relational data analysis in understanding team mechanisms. Practically, they suggest that optimizing rostering and zoning to enhance structural connectivity, fostering a psychologically safe climate to strengthen collaborative efficacy, promoting strategic competency development to build professional complementarity, and implementing structured mentoring to support experiential transmission may together offer an evidence‐based pathway to continuously improve collaboration quality in critical care nursing.

## Author Contributions

Jiaqi Shi: writing–original draft preparation, software, and methodology. Qingxia Quan: software and data curation. Kaili Ye: visualization and investigation. Lulu Feng: methodology and writing–reviewing and editing. Fangfang Chen: investigation and software. Jianghui Yang: investigation and writing–reviewing and editing. Zhenhong Fang: supervision and conceptualization.

## Funding

This research did not receive any specific grant from funding agencies in the public, commercial, or not‐for‐profit sectors.

## Conflicts of Interest

The authors declare no conflicts of interest.

## Supporting Information

Additional supporting information can be found online in the Supporting Information section.

## Supporting information


**Supporting Information** Supporting Table S1presents the full list of candidate variables considered during predictor selection, together with the rationale for inclusion or exclusion. Supporting Figure S1 illustrates the LASSO coefficient shrinkage paths and cross‐validation results used for predictor selection.

## Data Availability

The data that support the findings of this study are available from the corresponding author upon reasonable request.
