# Supplementary material for: Collaborative Networks in Critical Care Nursing Teams: Based on Social Network Analysis and Multiple Regression Quadratic Assignment Procedure
Source: J Nurs Manag. 2026 Jun 18;2026:6045308. doi: 10.1155/jonm/6045308 (PMC13278028; doi:10.1155/jonm/6045308)
Supplement: Supplementary file 1 — Supporting Information Supporting Table S1presents the full list of candidate variables considered during predictor selection, together with the rationale for inclusion or exclusion. Supporting Figure S1 illustrates the LASSO coefficient shrinkage paths and cross‐validation results used for predictor selection. [file JONM-2026-6045308-s001.docx]

Design summary: The initial candidate pool comprised 14 variables from structural, individual, relational, and competency domains. Expert consultation excluded 8 variables based on scientific relevance and feasibility. Six variables were shortlisted; after a final feasibility/collinearity screen, six were entered into LASSO with the collaboration matrix (Y) as the outcome. Variables retained by LASSO were included in the primary MRQAP model.

Supplement Table S1. Candidate explanatory variables and selection pathway

| Domain | Variable | Data source | Expert shortlist (Yes/No) | Entered LASSO (Yes/No) | Included in final model (Yes/No) | Reason for exclusion |
| --- | --- | --- | --- | --- | --- | --- |
| Structural | Work assignment proximity | Duty roster; responsibility units | Yes | Yes | Yes |  |
| Individual | Psychological safety similarity | Edmondson 7-item scale | Yes | Yes | Yes |  |
| Relational | Tenure similarity | HR records (ICU years) | Yes | Yes | Yes |  |
| Competency | Competency complementarity | Training & certification logs (ACLS, CRRT, ventilator, transfusion) | Yes | Yes | Yes |  |
| Individual | Informal subgroup co-membership | Sociometric name-generator; community detection | Yes | Yes | No | Excluded by lasso |
| Structural | Patient acuity overlap | Unit dashboard; patient-level acuity scores | Yes | Yes | No | Excluded by lasso |
| Structural | Spatial proximity | Bed/zone assignment in roster | No | — | No | Excluded at expert screen: redundant with work assignment; limited managerial actionability |
| Structural | Formal unit co-membership | Organizational records | No | — | No | Excluded at expert screen: overlaps with work assignment definitions |
| Competency | Shared certifications | Training & certification logs | No | — | No | Redundant with competency complementarity; limited additional value |
| Relational | Trust/consultation frequency | Brief survey | No | — | No | Excluded at expert screen: expected high missingness; overlaps with informal subgroup |
| Relational | Prior collaboration (lag) | Pre-observation collaboration logs | No | — | No | Excluded at expert screen: retrospective data unavailable |
| Relational | Triadic closure (common collaborators) | Derived from prior network | No | — | No | Excluded at expert screen: requires longitudinal network not available |
| Structural | Overtime overlap | Time-clock data | No | — | No | Excluded at expert screen: not essential to primary research question |
| Structural | Co-exposure to unit stress | Unit-day indicators (admissions, transports, codes, occupancy) | No | — | No | Excluded at expert screen: construct ambiguity; low added value |

Abbreviations: ICU, intensive care unit; ACLS, Advanced Cardiovascular Life Support; CRRT, Continuous Renal Replacement Therapy.


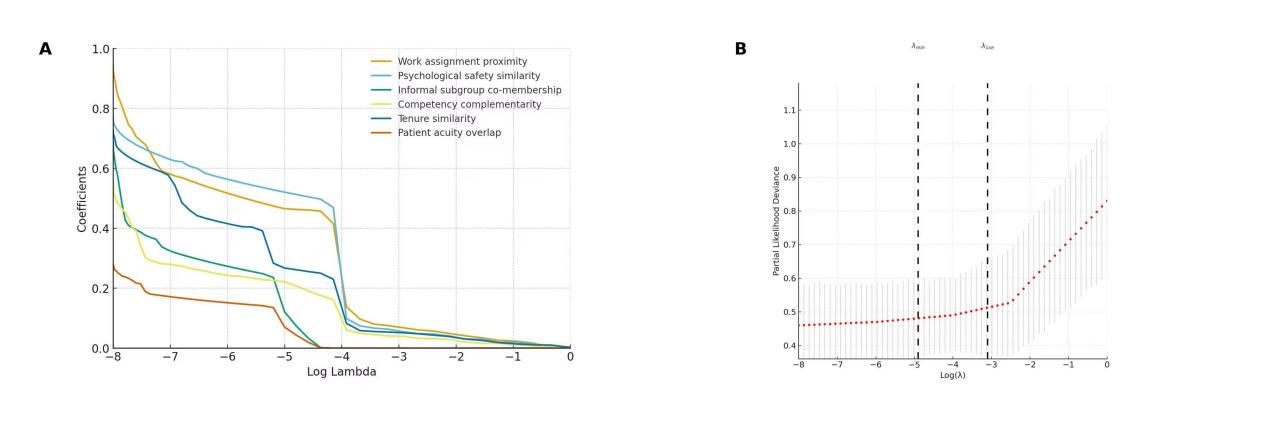


Supplement Figure S1. LASSO-based predictor selection for nurse-to-nurse collaboration.

Panel A: coefficient paths across log λ. Panel B: tenfold cross-validation;
